# Well-differentiated papillary mesothelial tumor of the peritoneum in a young woman: A case report with molecular insights

**DOI:** 10.1097/MD.0000000000046241

**Published:** 2025-11-28

**Authors:** Ren Xu, Shuo Xu, Xiaona Wang, Yanan Ren, Luyang Su, Jianzhi Su

**Affiliations:** a Department of Gynecology, Hebei General Hospital, Shijiazhuang, China; b Physical Examination Center, Hebei General Hospital, Shijiazhuang, China; c Department of Urology Surgery, The Fourth Hospital of Hebei Medical University, Shijiazhuang, China.

**Keywords:** diagnosis, immunohistochemistry, next-generation sequencing, peritoneal mesothelioma, well-differentiated papillary mesothelioma

## Abstract

**Rationale:**

Well-differentiated papillary mesothelial tumor (WDPMT) is an uncommon, slow-growing neoplasm that is often an incidental finding in the peritoneum of women of reproductive age. It can be misdiagnosed as malignant mesothelioma on routine histology, potentially leading to unnecessary aggressive therapy. The role of modern molecular techniques in resolving this diagnostic dilemma and guiding conservative management warrants emphasis.

**Patient concerns:**

A 32-year-old woman presented with vague abdominal discomfort. Imaging revealed bilateral ovarian cysts and significant pelvic free fluid.

**Diagnoses:**

Laparoscopy identified multiple small peritoneal nodules. Initial frozen-section histology suggested malignant mesothelioma. Definitive diagnosis of WDPMT was established through comprehensive immunohistochemistry (retained BAP1 expression) and next-generation sequencing, which revealed a low tumor mutational burden and a pathogenic GPR124 mutation. This genetic profile distinct from malignant mesothelioma.

**Interventions:**

After definitive diagnosis and thorough counseling, the patient opted against cytoreductive surgery or systemic therapy. A strategy of active surveillance with serial imaging and tumor marker assessment was implemented.

**Outcomes:**

The patient remained asymptomatic with no evidence of disease recurrence or progression 24 months after diagnosis.

**Lessons:**

This case highlights that a definitive distinction between WDPMT and malignant mesothelioma is paramount, as it dictates a radically different management strategy. Integration of immunohistochemistry (particularly BAP1) and molecular profiling is essential for accurate diagnosis and can prevent overtreatment. For appropriately selected patients with WDPMT, conservative management with active surveillance represents a safe and effective approach, underscoring the value of precision medicine in guiding patient-centric care.

## 1. Introduction

Well-differentiated papillary mesothelial tumor (WDPMT), previously termed well-differentiated papillary mesothelioma, is a rare, distinct entity characterized by papillary architecture lined by bland mesothelial cells, typically lacking stromal invasion. It most commonly arises incidentally in the peritoneum, particularly in women of reproductive age, though rare cases occur in the pleura, pericardium, or tunica vaginalis.^[1–3]^ Historically, its relationship to malignant mesothelioma was debated, with uncertainty regarding whether it represented a reactive process, a benign neoplasm, or a precursor to malignancy.^[4]^ Advances in molecular pathology have been pivotal in resolving this ambiguity. Comprehensive genomic analyses now definitively demonstrate that WDPMT is genetically distinct from malignant mesothelioma (both peritoneal and pleural).^[5,6]^ WDPMT harbors frequent mutually exclusive mutations in TRAF7 or CDC42, exhibits a characteristic enrichment of C > A transversion mutations and COSMIC mutational signature 24, and crucially lacks the hallmark alterations of malignant mesothelioma (e.g., BAP1, CDKN2A/B, NF2, and SETD2 inactivation).^[7]^ Consequently, the World Health Organization classification now designates it as a separate tumor of low malignant potential or uncertain behavior, emphasizing its generally indolent clinical course.^[8]^

This case report details a diagnostically challenging presentation of peritoneal WDPMT in a 32-year-old woman, underscoring critical aspects of this rare tumor. Initially misdiagnosed as epithelioid malignant mesothelioma based on morphology and limited immunohistochemistry (IHC) of incidentally discovered peritoneal nodules during laparoscopy for an ovarian cyst, the case highlights the significant risk of misclassification without comprehensive evaluation.^[9]^ The definitive diagnosis relied on expert pathology review integrating expanded IHC (confirming retained BAP1 expression and specific mesothelial markers) and next-generation sequencing, which revealed a low tumor mutational burden and a GPR124 mutation (variant allele frequency 2.1%)—a finding not commonly reported in WDPMT genomic studies. This case is unique in demonstrating the essential role of specialized IHC and molecular profiling in differentiating WDPMT from its malignant mimics, especially in young patients. Furthermore, it exemplifies the typically favorable prognosis associated with WDPMT; consistent with large cohort studies showing no disease-specific mortality and no proven benefit for aggressive cytoreduction or systemic therapy, the patient remains asymptomatic and recurrence-free under surveillance alone 20 months post-diagnosis. The incidental discovery, initial diagnostic pitfalls, utilization of minimally invasive single-port laparoscopy, and successful conservative management align with the evolving understanding of WDPMT as a molecularly defined entity with indolent biological behavior.^[10]^

## 2. Case presentation

A 32-year-old nulliparous woman was admitted on October 9, 2023, for evaluation of incidentally discovered bilateral ovarian cysts and pelvic fluid first noted 7 months prior on routine ultrasound. Serial ultrasounds revealed a persistent, complex left ovarian cyst (max dimension ~5.6 cm) with internal echoes and wall irregularities, heterogeneous uterine myometrial echoes, and significant pelvic fluid (max depth ~5.3 cm). She reported no menstrual changes, vaginal bleeding or discharge, abdominal pain, or nausea and vomiting. The initial tumor markers showed normal levels of HE4 (62.14 pmol/L) and CA125 (19.06 U/mL), while CA199 was significantly elevated (301.2 U/mL). Upon admission, all these markers normalized (HE4: 48.61 pmol/L, CA199: 14.46 U/mL, CA125: 17.73 U/mL). Gynecological examination revealed a retroverted, mobile uterus and left adnexal thickening.

## 3. Treatment

**Figure 1.**
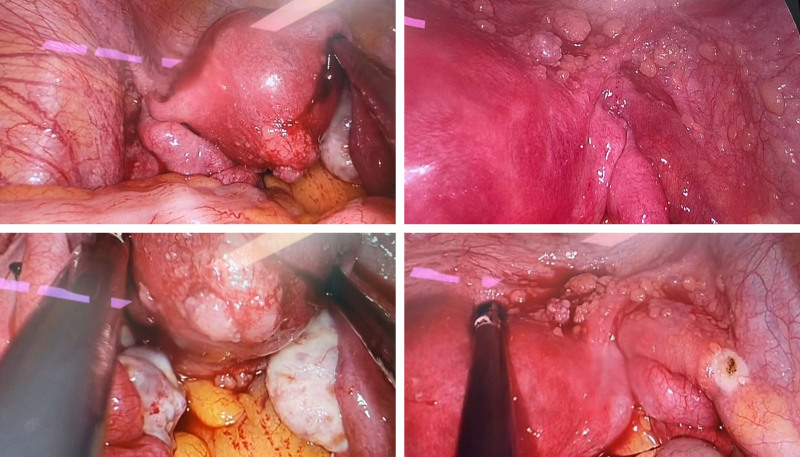
Intraoperative view during laparoscopy.

As shown in Figure 1, diagnostic laparoscopy (October 12, 2023) revealed a normal uterus, a smooth 7-cm left ovarian cyst without adhesions, and the critical finding of diffuse, scattered millet-like nodules (0.5–1.0 cm) involving the pelvic peritoneum (sidewalls, bladder reflection, uterine serosa, and rectouterine pouch). No disease was seen on the omentum, bowel, liver, or diaphragm. Frozen-section analysis of biopsied peritoneal nodules suggested a malignant papillary epithelioid tumor, while the resected ovarian cyst appeared benign. The patient elected for cystectomy and biopsy only, deferring definitive peritoneal surgery pending final pathology.

**Figure 2.**
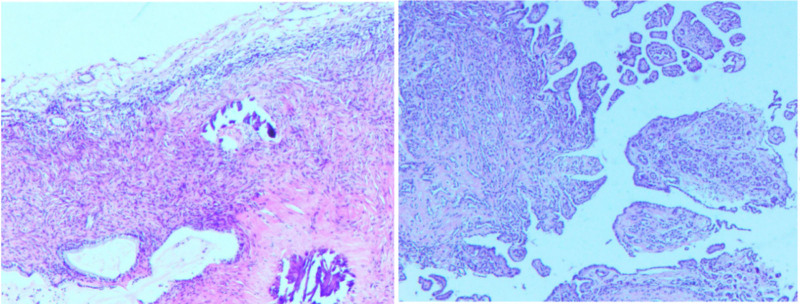
Histopathological examination of the surgical specimen.

The patient underwent single-port laparoscopic left ovarian cystectomy and biopsy of representative right pelvic peritoneal nodules. Figure 2 shows the final pathology from the primary institution: left ovarian cyst wall—mucinous cystadenoma with multifocal calcification. Peritoneal nodule—mesothelial-origin tumor, immunohistochemistry consistent with epithelioid malignant mesothelioma (IHC: CKpan+, CR+, CK5/6+, partial D2–40+, WT-1+, EMA+, focal Glut-1+, hotspot Ki-67 ~10%). Genetic testing is recommended. The discharge diagnoses were thus Peritoneal Mesothelioma and Left Ovarian Mucinous Cystadenoma.

Critical diagnostic refinement came from expert pathology consultation at Peking University Third Hospital. Review of the peritoneal lesion, integrating morphology, the original IHC, additional specialized IHC (HBME-1 partial+, CK17+, PAX8+, BAP1+, L1CAM‐, P16 partial+, and MTAP+), and next-generation sequencing results (GPR124 mutation, variant allele frequency 2.1%; low tumor mutational burden 0 mut/Mb; MSS), established the definitive diagnosis: WDPMT. The ovarian cyst pathology was also revised to Endometriotic cyst with mucinous metaplasia and focal calcification, consistent with endometriosis.

Given the confirmed diagnosis of WDPMT—a distinct entity with indolent biological behavior compared to malignant mesothelioma—and after comprehensive counseling regarding the prognosis and management options (including observation, complete cytoreductive surgery, or systemic therapy), the patient opted against further surgical resection of the remaining peritoneal nodules or adjuvant therapy. A strategy of close clinical surveillance was implemented. As of the last follow-up on June 14, 2025 (approximately 20 months post-diagnosis), the patient remains asymptomatic with no clinical or radiological evidence of disease recurrence or progression, aligning with the typically favorable course of WDPMT under conservative management.

## 4. Discussion

WDPMT is a rare neoplasm, and recent advancements in molecular genetics have provided important insights into its pathogenesis, clinical features, and management. The genetic landscape of WDPMT is distinct from that of malignant mesotheliomas, which has led to a more refined understanding of this disease entity. Studies by Yu et al and Shrestha et al have identified specific somatic mutations in genes such as E2F1, EHD1, and ATM, which contribute to the pathogenesis of WDPMT.^[1,11]^ For example, the E2F1 mutation (R166H), identified by Yu et al, is located in the conserved DNA-binding domain, disrupting the protein’s ability to bind DNA and activating downstream target genes.^[11]^ This mutation also results in increased protein stability, potentially contributing to the development of WDPMT. These findings underline the importance of genetic alterations in the pathogenesis of WDPMT and distinguish it from malignant mesotheliomas, reinforcing the notion that WDPMT is a distinct neoplasm with its own genetic signatures.

An intriguing clinical aspect of peritoneal WDPMT is its well-documented association with endometriosis—a finding also present in our case, which involved an endometriotic cyst. This frequent co-occurrence is supported by several clinical studies, including a large case series by Malpica et al, which reported endometriosis in up to 23% of peritoneal WDPMT.^[6]^ Haba et al described a 48-year-old woman who underwent surgery for adenomyosis, during which multiple papillary nodules were discovered on the pelvic serosa; postoperative pathological examination confirmed WDPMT.^[12]^ Similarly, Mangal et al reported a case of WDPMT diagnosed incidentally during surgery for recurrent endometriosis.^[13]^ Collectively, these cases suggest that chronic inflammation or repair processes may provide a pathological microenvironment conducive to the development of WDPMT.^[14]^ They also underscore the importance of maintaining a high index of suspicion for atypical peritoneal lesions and performing biopsies during surgery for endometriosis. Regarding the malignant potential of WDPMT, several case reports have raised concerns about its ability to undergo malignant transformation. Washimi et al and Costanzo et al documented rare cases of WDPMT that eventually transformed into diffuse malignant mesothelioma after several years.^[15,16]^ These reports highlight the need for long-term surveillance of patients with WDPMT, despite its generally indolent nature. In the case reported by Washimi et al, the malignant transformation occurred after 7 years, emphasizing that WDPMT, though usually benign, can rarely evolve into a more aggressive disease.^[15]^ Similarly, Costanzo et al observed malignant transformation 13 years after the initial diagnosis, further advocating for close monitoring.^[16]^ However, it is important to note that such transformations are rare, and most patients with WDPMT experience a favorable clinical course.

From a therapeutic standpoint, WDPMT is primarily managed with surgical resection. As emphasized by Vogin et al and Sun et al, WDPMT is often diagnosed incidentally during surgery for other conditions.^[17,18]^ Surgical intervention, typically cytoreductive surgery, remains the cornerstone of treatment. Although there is no consensus on the role of chemotherapy or radiotherapy for WDPMT, some studies suggest that these modalities might be considered in cases with recurrent or diffuse disease. Washimi et al proposed that hyperthermic intraperitoneal chemotherapy could be beneficial in patients with advanced, recurrent disease, although evidence supporting this approach is limited.^[15]^ The lack of response to chemotherapy and radiation in most WDPMT cases suggests that the indolent biological behavior of the tumor is a significant factor in its management.^[19]^

The location of WDPMT nodules—whether confined to the peritoneum or involving the ovary—may influence the surgical approach but does not fundamentally alter the overall management philosophy. When WDPMT involves the ovary, it is often superficial and can be managed with ovarian cystectomy or partial resection, similar to peritoneal nodules. In such cases, the primary goal remains obtaining a definitive diagnosis while preserving ovarian function, particularly in young women. Importantly, ovarian involvement by WDPMT does not imply a more aggressive biology, and there is no evidence that it necessitates more extensive surgery or adjuvant therapy compared to purely peritoneal disease. Thus, even with ovarian involvement, conservative surgery followed by surveillance remains a reasonable strategy, consistent with the indolent behavior of WDPMT.

In terms of prognosis, WDPMT is associated with an excellent long-term survival rate. Vogin et al confirmed a median disease-free survival of 144 months in a cohort of 56 patients, underscoring the indolent nature of the disease.^[17]^ Most WDPMT cases exhibit no signs of tumor progression or local recurrence after surgical intervention, and survival rates remain high even without aggressive treatment. Sun et al further highlighted the role of PAX8 immunohistochemistry as a sensitive and specific marker for distinguishing WDPMT from malignant mesotheliomas, aiding in the accurate diagnosis and appropriate management of the disease.^[18]^ The absence of BAP1 and MTAP/CDKN2A expression in WDPMT, as shown by Hassan et al, also plays a crucial role in differentiating it from mesothelioma in situ, which has a different clinical course and potential for malignancy.^[20]^

In conclusion, WDPMT represents a distinct clinical and molecular entity with a relatively benign prognosis, though careful monitoring is warranted given the potential for rare malignant transformation. Genetic profiling, particularly the identification of mutations in genes such as E2F1, TRAF7, and CDC42, provides crucial information for understanding the disease’s pathogenesis. Despite its indolent nature, the management of WDPMT requires careful consideration of surgical options and long-term surveillance, especially in cases with recurrent or symptomatic disease. The prognosis is generally favorable, with most patients experiencing prolonged survival post-surgery.

## 5. Conclusion

This case highlights the diagnostic complexities and clinical significance of accurately distinguishing WDPMT from malignant mesothelioma. Despite initial concerns of malignancy, comprehensive immunohistochemistry and next-generation sequencing ultimately confirmed WDPMT—a low-grade, indolent neoplasm with a favorable prognosis. This reinforces the critical role of molecular diagnostics in avoiding overtreatment, especially in young patients. Furthermore, the successful use of conservative management and continued disease-free status after 20 months emphasize that, in properly diagnosed WDPMT, surveillance can be a safe and effective strategy. Clinicians should remain vigilant for rare malignant transformations, but also recognize that most cases follow a benign course. This case exemplifies how precision diagnostics can guide individualized patient care while minimizing unnecessary interventions.

## Acknowledgments

The authors would like to express their gratitude to the participants who participated in the study.

## Author contributions

**Formal analysis:** Ren Xu, Shuo Xu.

**Resources:** Ren Xu, Shuo Xu.

**Writing – original draft:** Ren Xu, Shuo Xu, Xiaona Wang, Yanan Ren, Luyang Su, Jianzhi Su.

**Writing – review & editing:** Jianzhi Su.
